# Targeted cell delivery of mesenchymal stem cell therapy for cardiovascular disease applications: a review of preclinical advancements

**DOI:** 10.3389/fcvm.2023.1236345

**Published:** 2023-08-04

**Authors:** Carlos Theodore Huerta, Francesca A. Voza, Yulexi Y. Ortiz, Zhao-Jun Liu, Omaida C. Velazquez

**Affiliations:** ^1^ DeWitt Daughtry Family Department of Surgery, University of Miami Miller School of Medicine, Miami, FL, United States; ^2^ Vascular Biology Institute, University of Miami Miller School of Medicine, Miami, FL, United States; ^3^ Department of Biochemistry & Molecular Biology, University of Miami Miller School of Medicine, Miami, FL, United States

**Keywords:** targeted cell delivery, mesenchymal stem cell, stem cell therapy, cell-based therapy, cardiovascular disease, chronic limb-threatening ischemia, myocardial infarction

## Abstract

Cardiovascular diseases (CVD) continue to be the leading cause of morbidity and mortality globally and claim the lives of over 17 million people annually. Current management of CVD includes risk factor modification and preventative strategies including dietary and lifestyle changes, smoking cessation, medical management of hypertension and cholesterol lipid levels, and even surgical revascularization procedures if needed. Although these strategies have shown therapeutic efficacy in reducing major adverse cardiovascular events such as heart attack, stroke, symptoms of chronic limb-threatening ischemia (CLTI), and major limb amputation significant compliance by patients and caregivers is required and off-target effects from systemic medications can still result in organ dysfunction. Stem cell therapy holds major potential for CVD applications but is limited by the low quantities of cells that are able to traffic to and engraft at diseased tissue sites. New preclinical investigations have been undertaken to modify mesenchymal stem cells (MSCs) to achieve targeted cell delivery after systemic administration. Although previous reviews have focused broadly on the modification of MSCs for numerous local or intracoronary administration strategies, here we review recent preclinical advances related to overcoming challenges imposed by the high velocity and dynamic flow of the circulatory system to specifically deliver MSCs to ischemic cardiac and peripheral tissue sites. Many of these technologies can also be applied for the targeted delivery of other types of therapeutic cells for treating various diseases.

## 1. Introduction

The efficacy of cell-based therapeutics, such as those utilizing mesenchymal stem/stromal cells (MSCs), is contingent upon effective cellular engraftment and homing to disease sites in order to re-establish homeostasis and enact their therapeutic benefits. Local injection to the site of disease or intravascular delivery are the most commonly utilized methods of administration of stem cells—including intraarterial injection that quickly delivers therapeutic cells to the tissue/organs fed by a given artery, and intravenous routes, by which therapeutic cells are infused into the bloodstream (systemic delivery) (1, 2). Unfortunately, these delivery routes are associated with substantial limitations. Direct injection is a popular method of administration particularly in sites with poor systemic perfusion; however, cell-based therapy is significantly limited by cell viability and retention even when locally dispensed to sites of disease (1, 3). Moreover, cells delivered to local disease sites may not function appropriately given environmental factors at the tissue interface including poor blood flow, hyperglycemia, hypoxia, and wide-spread local inflammation associated with disease (4–7). Limited space and even physical pressure at sites of inoculation may further diminish cellular characteristics or hamper cell viability (5, 8, 9). However, many disease sites are not amenable to access by local inoculation given their intra-cavitary anatomic location (cardiothoracic organs, brain, aorta, etc.), which would necessitate highly invasive injection procedures. Systemic administration via intravenous routes can partially overcome this limitation in delivery to locations that are traditionally hard to access. Unfortunately, this can lead to indiscriminate trafficking of cells and often results in a low number of cells delivered to anatomic locations afflicted by disease (1, 10). As a result, there is a critical, unmet need for novel methods to augment delivery of a sufficient quantity of therapeutic cells to diseased tissue sites.

**Figure 1:**
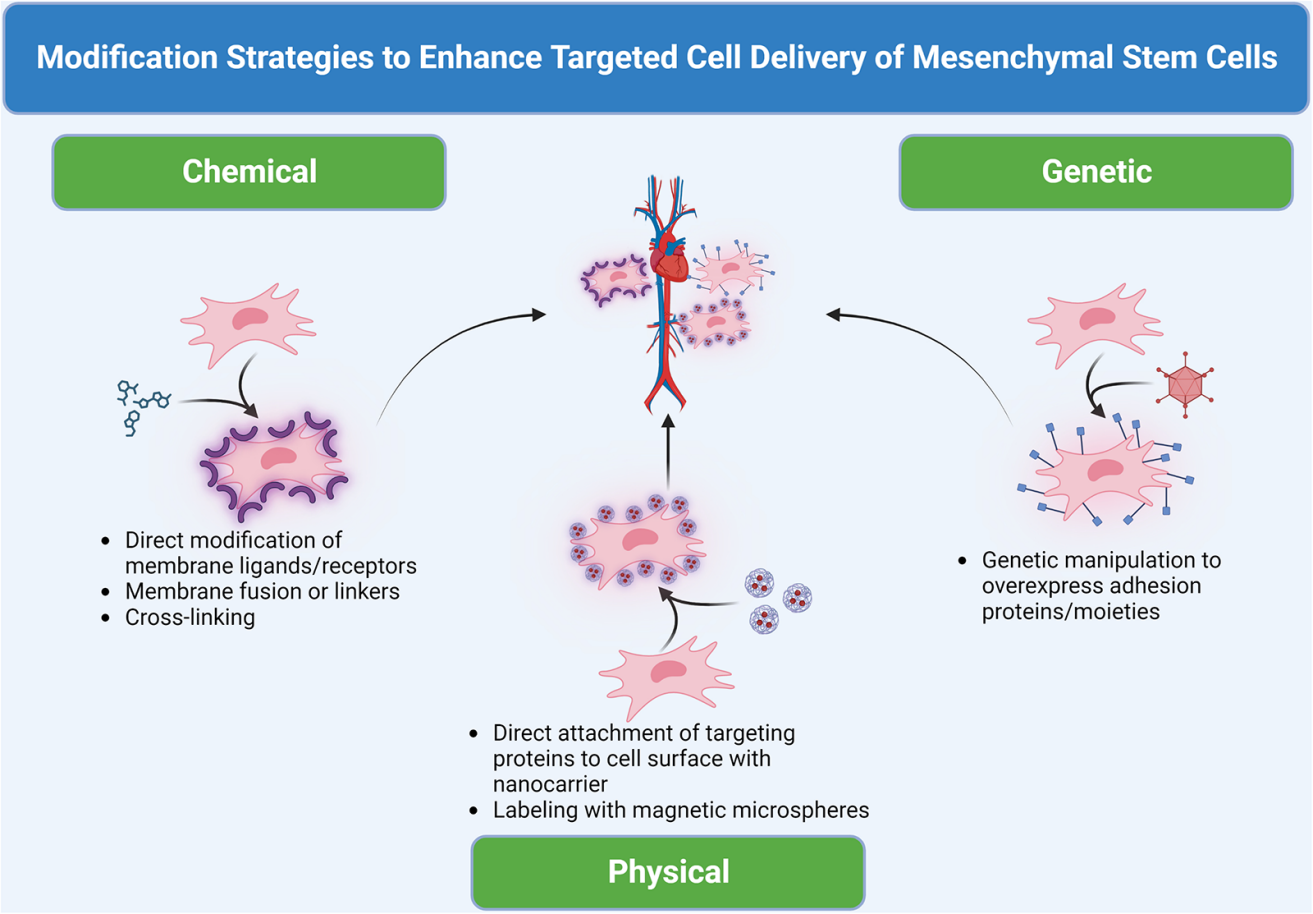
Domains of modification of therapeutic MSCs for targeted cell delivery to cardiovascular sites. Three methods, including chemical, physical and biological, can be used to modify the cell surface or label cells to create or install “GPS”/“sensor” and guide cell homing.

A novel area of investigation within the field of cell-based therapeutics is the advent of targeted cell delivery to limit the downsides of systemic cell administration as well as enhance cell therapeutic efficacy. The purpose of this review is to summarize the most recent preclinical advances in the arena of targeted cell delivery for applications in cardiovascular and peripheral vascular disease applications achieved through physical, chemical, and genetic modifications to enhance the delivery of MSCs (Figure 1).

## 2. Basis for modifying MSCs for targeted cell delivery in cardiovascular applications

At the circulatory system interface, endothelial cells form a selectively permeable luminal barrier between blood and tissues under homeostatic conditions. As a result, unstimulated/unactivated healthy endothelium in normal tissue forms a smooth lining that contributes to the laminar flow of the circulatory system and facilitate the transport of cells and nutrients to peripheral sites. After physiologic insult from tissue injury, inflammation, and/or malignancy, numerous chemokines and cytokines such as stromal cell-derived factor 1 alpha (SDF-1α) and transforming growth factor beta (TGF-β) activate neighboring endothelial cells (11, 12). Resultant stimulation by a cytokine milieu specific to each pathologic state can cause a variety of cell adhesion molecules (CAMs) such as integrins and selectins to be highly expressed within the luminal endothelium in the diseased tissue; thereby, making the endothelial lining “sticky” and augmenting its ability to tether circulating cells, such as endothelial progenitor cells (EPCs), MSCs, and leukocytes, responding to tissue repair and inflammatory signals (12). As a result, methods to exploit cognate protein-receptor binding interactions between circulating MSCs and activated endothelial cells at sites of ischemic vasculature after myocardial infarction (MI) or peripheral ischemic insults can dramatically augment the ability of MSCs to traffic to diseased tissue sites.

**Figure 2:**
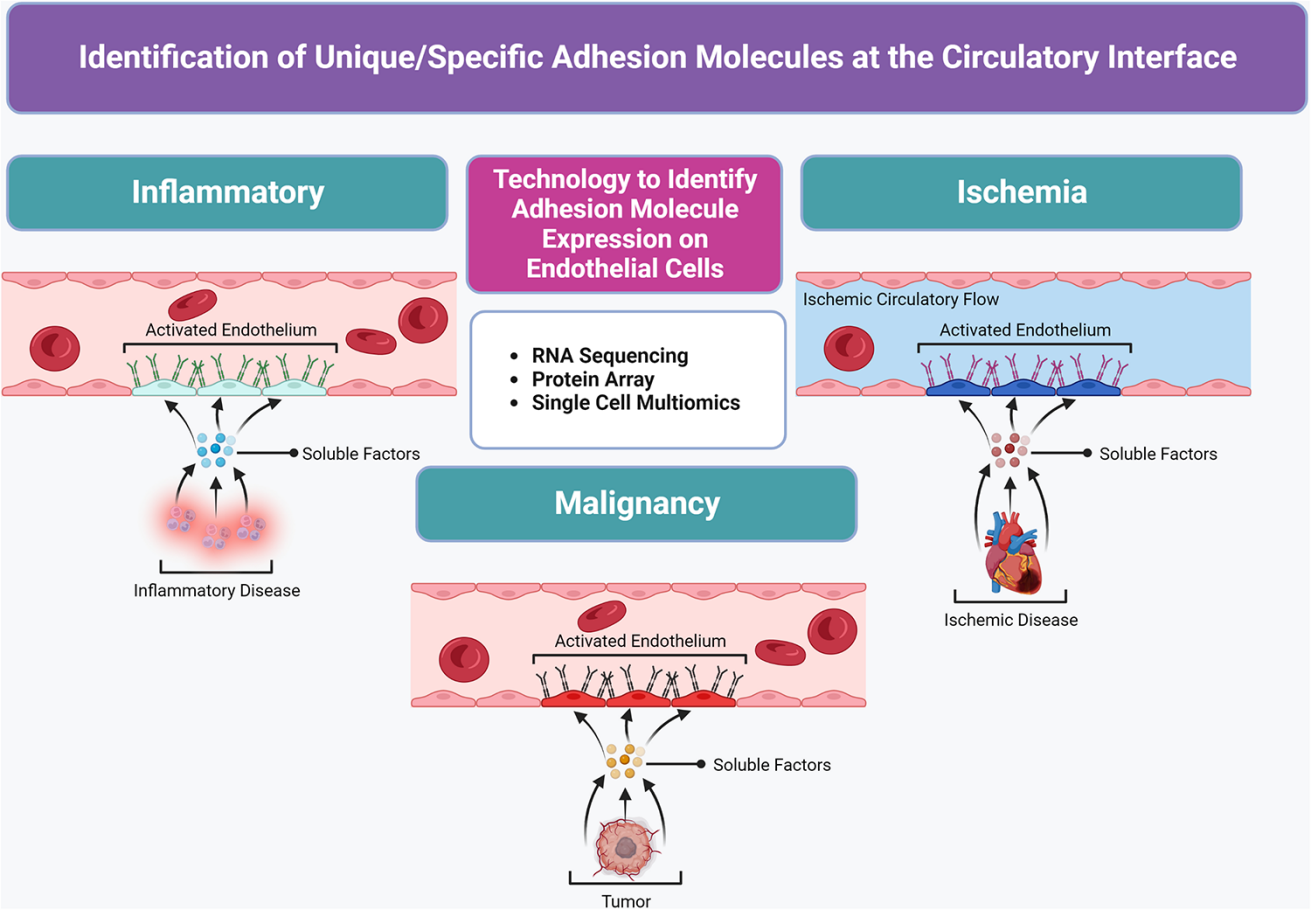
Molecular profiling advancements used to identify unique/special target(s) at the circulatory system interface in diseased tissues to create a “roadmap” for the targeted delivery of therapeutic MSCs. Unique cytokine/chemokine milieu in various diseased tissue states stimulate local vasculature to express a panel of elevated or specific adhesion molecules in activated endothelium.

Ultimately, the cytokine profile and subsequent endothelial cell surface molecules induced are unique to the underlying inflammatory, ischemic, or oncologic pathophysiology (Figure 2). To better characterize highly upregulated target CAMs on endothelial sites in diseased tissues, the utilization of newly available RNA sequencing, protein array technology, and single-cell multiomics has widely propagated (13–15). For example, Fournier et al. (16) utilized bulk RNA sequencing of meningeal endothelial cells to identify upregulation of coxsackie- and adenovirus receptor-like membrane protein (CLMP) as a trafficking molecule upregulated by inflammation in neurologic conditions such as multiple sclerosis. They further demonstrated that overexpressed CLMP is utilized by immune cells to traffic across endothelial cells into the central nervous system to modulate neuroinflammation, and this CLMP overexpression could represent a new therapeutic target for modified cell therapies to exploit and target the central nervous system (16). The continued applications of similar profiling methods will help to uncover unique adhesion molecules expressed on endothelial cells at specific disease sites in order to target their cognate ligands as molecular mechanisms and augment the targeted cell delivery of stem cells for cardiovascular applications in future work.

## 3. Physical methods of guiding cell engraftment

With the advent of novel molecular and synthetic platforms, new avenues of manipulating the cell surface to change migratory patterns of cells in circulation have become available to researchers. Such methods offer the ability to physically alter or decorate the cell surface with specific proteins without aberrant changes to intrinsic MSC properties that can occur through genetic manipulation or downstream cell signaling (7). Nanocarriers utilizing adhesion moieties or cell membrane components have emerged as a versatile, safe method to achieve the delivery of drug and cell therapeutic payloads to specific tissue sites expressing the cognate receptor for proteins on a modified nanocarrier (5, 17–19). For instance, E-selectin, an inducible CAM with pro-angiogenic signaling effects, along with its ligands, including CD44, CD62 and E-selectin ligand (ESL), have been shown to be upregulated on vasculature in ischemic tissue (20, 21). Liu et al. (5) demonstrated that systemic administration of pre-modified MSCs on which cell surfaces were coated with E-selectin-conjugated dendrimer nanocarriers directed MSC homing to skin wound sites or injured cornea mediated by enhanced interaction of E-selectin/ligands. This resulted in increased tissue repair in an ischemic diabetic wound healing model. This physical cell modification led to efficient trafficking of MSCs to wound areas without indiscriminate distribution to other organs even when administered systemically (5).

Through an extension of the beneficial angiogenic effects of the numerous cell signaling and paracrine factors secreted by MSCs, some groups have created nanocells by combining nanocarrier/membrane derivatives with the MSC secretome. By exploiting the overexpression of prostaglandin E2 (PGE2) receptors in ischemic myocardium, Su et al. (22) designed a platelet-inspired nanocell (PINC) containing PGE2-platelet membranes and cellular signaling factors harvested from the cardiac stem cell (CSC) secretome. Intravenous administration of PGE2-PINCs achieved targeted delivery of the CSC-derived therapeutic payload to the injured myocardium in a murine model of myocardial ischemia and reperfusion injury. This therapy significantly mitigated ischemia and reperfusion injury in myocardium as evidenced by preserved cardiac ejection fraction (EF), increased cycling of cardiomyocytes, activation of endogenous CSCs, and enhanced angiogenesis in cardiac tissue *in vivo* compared to control PINC treatment lacking PGE2-platelet membranes (22). This work demonstrates a new method to direct the MSC therapeutic payload to target sites.

Some have even investigated the application of physical devices to guide stem cell transplantation in difficult locations. For instance, the retention of stem cells to the heart is often impeded due to venous washout resulting from forceful cardiac contraction even when delivered via direct intracoronary injection (23). Hydrogel and microgel formulations as well as direct cell coating are applications that have been extensively investigated in murine and porcine models of myocardial ischemia to overcome challenges in cell retention. Utilizing an alginate scaffold to aid the local implantation of MSCs in a porcine model of MI, Panda et al. (24) demonstrated substantially preserved cell retentiona as well as improved cardiac impulse condution. Others such as Peng et al. (25) have utilized a gelatin methacrylate cell-coating system on murine-derived MSCs locally inoculated to the heart after induction of MI. Cell coating with gelatin methacrylate offered a less bulky matrix compared to conventional hydrogels for MSCs to more directly interact with diseased tissue sites, which resulted in preserved cardiac function, reduced scar size, and augmented angiogenesis compared to control treatments (25). While biomaterials such as MSC-loaded scaffolds and devices hold promise, they still incur postoperative complications associated with implantation of medical devices such as fibrosis and adhesions that can complicate procedures later in life (23). To overcome these challenges, Cheng et al. (26) investigated the use of CSCs labeled with iron microspheres. After ligation of the left anterior descending artery to simulate myocardial ischemia in rats, administration of iron labeled-CSCs via left ventricular injection occurred and was followed by the placement of an external magnet outside the heart for 10 min. Overall, rats exposed to the external magnet demonstrated substantially augmented rates of stem cell engraftment in ischemic heart tissue and significantly reduced migration of therapeutic cells to the lungs. These effects persisted up to 3 weeks after injection and resulted in enhanced therapeutic benefits including higher ventricular EF and attenuated left ventricular remodeling compared to rats not exposed to the external magnet device during stem cell administration (26). Similarly, Shen et al. (27) utilized rat MSCs labeled with superparamagnetic oxide nanoparticles (SPIOs) administered into the left ventricle of recipient rats after induction of myocardial ischemia and placed magnets of varying intensity at the cardiac site. They importantly found that cellular engraftment was significantly correlated with higher magnetic intensity; however, the highest magnetic intensity resulted in microembolization and undermined the clinical benefits of cell therapy. Ultimately, these works serve as examples of physical devices to potentiate cell delivery and retention at target disease tissues without the risks associated with the implantation of long term hardware. While physical alterations such as these hold significant potential to improve targeted cell delivery for cardiovascular applications, future studies will need to consider and rigorously test the safety and toxicity associated with nanocarriers and other synthetic platforms (5, 17). Furthermore, physical devices to guide stem cell engraftment are a unique way to enhance cell transplantation but will need to optimize the least invasive methods possible of device utilization as well as consider their cost and scalability for clinical applications (26, 28).

## 4. Genetic manipulations to direct cell migration

While physical methods of manipulating the cell surface of MSCs can augment their migratory ability, newer methods of genetic manipulation have emerged as a promising method to overexpress cell-cell binding proteins for delivery and further potentiate the innate properties and therapeutic effects of MSCs (29, 30). For example, colony-stimulating factor 2 (CSF2) is a signaling chemokine that along with its cognate receptor pair CSF2RB modulates leukocyte production and proliferation as well as MSC recruitment and post-myocardial infarction (MI) cardiac remodeling (31). However, expression of CSF2RB is very low in unmodified MSCs (32, 33). To overcome this, Qi et al. (32) utilized an adenovirus vector to induce overexpression of CSF2RB in adipose-derived MSCs (AD-MSCs) prior to intravenous delivery in murine subjects after myocardial ischemia and reperfusion injury. Compared to unmodified AD-MSCs, CSF2RB-modified AD-MSCs became imbued with a cardioprotective phenotype that resulted in markedly preserved ventricular EF and cardiac contractility in injured mice. Moreover, genetic modification with CSF2RB augmented AD-MSCs’ proangiogenic properties and inhibited cell apoptosis in addition to increasing their migratory capacity through increased phosphorylation of STAT5 within the canonical JAK-STAT5 signaling pathway. Modification of MSCs in this way to improve their migratory capacity after intravenous administration can also allow for repeated rounds of noninvasive treatment with cell delivery, thereby overcoming traditional drawbacks associated with invasive intramyocardial injections (34, 35). In a similar fashion, Yan et al. (36) implemented an adenoviral vector to induce overexpression of N-cadherin, a transmembrane cell-cell adhesion protein, in murine derived AD-MSCs. Cells harboring abundant cell surface N-cadherin levels demonstrated improved adhesion on to cardiomyocytes and paracrine effects on angiogenesis mediated by subsequent upregulation of matrix metalloproteinases (MMPs)-10 and -13 as well as hepatocyte growth factor (HGF). Furthermore, this cell therapy resulted in enhanced engraftment at ischemic myocardium and attenuated fibrosis after cardiac ischemia *in vivo*.

Parallel applications of genetic programming of MSCs have also been undertaken for peripheral vascular diseases such as chronic limb-threatening ischemia (CLTI) in recent years. Hematopoietic stem cells have previously been shown to express a CD44 glycoform known as hematopoietic cell E-/l-selectin ligand (HCELL), which functions as a highly potent ESL to improve their recruitment within bone marrow compartments (19). Modification of cell surface CD44 on stem cells into HCELL has been previously shown to enhance the tropism of these cells to bone, which can also provide a “roadmap” or “GPS” for targeted cell delivery to areas with heightened ESL expression such as in ischemic vasculature (18, 37, 38). To exploit this relationship with E-selectin, a CAM capable of inducing postnatal neovascularization, in ischemic tissue, Quiroz et al. (30) demonstrated a novel application of murine bone marrow-derived MSCs (BM-MSCs) for CLTI after inducing the overexpression of cell surface membrane-bound E-selectin via an adenoviral vector. By simulating limb ischemia via surgical ligation of the femoral artery, mice receiving local therapy with E-selectin-modified BM-MSCs demonstrated rescued revascularization, muscle fiber integrity, and ambulatory capacity on treadmill testing compared to control BM-MSC treatment (30). Interestingly, genetic modification of BM-MSCs with E-selectin also enhanced the angiogenic profile of these cells *in vitro* through the upregulation of nine proangiogenic genes such as *Cxcl2*, which among many functions also contributes to cell engraftment (30). Taken together, these studies show hope for augmenting not only cellular delivery and engraftment but also the cell potency of MSCs for future cardiovascular and peripheral disease applications. As researchers continue to explore such options for gene editing, they should consider the associated risks of these approaches such as aberrant insertional mutagenesis and chromosomal instability (39). Although viral vectors have been used in previous clinical applications, adverse immune reactions can occur with their use and impede the stability of gene expression (40, 41).

## 5. Chemical methods to potential cell delivery

Direct chemical modification of the cell surface can also yield improvements in cell trafficking. Although AD-MSCs offer advantages as a readily accessible tissue source of MSCs with potent paracrine and tissue reparative functions, their homing potential for cardiovascular purposes is significantly limited in unmodified cells (42–44). To overcome this challenge and allow targeting to denuded endothelial sites after vascular injury, Yan et al. (45) altered AD-MSCs utilizing a polyethylene glycol-conjugated derivative linker to facilitate the binding of P-selectin binding peptide (PBP) to the cell surface. After both wire and balloon injury to femoral artery sites in rats to simulate injury from percutaneous interventions observed in human patients, PBP-modified AD-MSCs targeted in significantly greater quantities to vascular injury sites after intraarterial injection and shielded injury sites from pathologic platelet and leukocyte adhesion. In turn, this substantially augmented vascular repair, limited pathologic neointimal hyperplasia, and augmented endothelial cell proliferation compared to unmodified AD-MSCs (45).

Chemical modification has similarly been applied to fuse components of other cell types with natural vascular homing abilities to MSCs. For instance, Tang et al. (46) harvested nanovesicles from platelets and fused them to the cell membrane of CSCs using polyethylene glycol. By exploiting the natural ability of platelet membranes in localizing to ischemic vascular sites and denuded endothelium, platelet-nanovesicle-fused CSCs augmented migration and retention to ischemic myocardium after intravenous delivery in a porcine model of cardiac ischemia and reperfusion (46). Moreover, this modified cell therapy yielded myocyte proliferation and angiogenesis in cardiac sites to a degree far greater than administering unmodified CSCs. As newer pharmacologic and chemical synthetic mechanisms continue to be developed, investigators will have a greater armamentarium of methods to modify MSCs for cardiovascular applications than ever before. However, limitations associated with chemical priming and modification strategies should be taken into account. For example, chemical agents can produce off-target, unintended effects on MSCs and result in transient expression of the molecular target (47). Additionally, scalability and manufacturing of sufficient quantities of MSCs using costly chemical strategies can pose a significant challenge in future clinical applications (48).

## 6. Host factors affecting modification strategies

While physical, chemical, and genetic modification of MSCs hold significant promise to augment their targeted delivery to disease tissues, the host milieu to which cells are delivered plays an equally important role in determining their therapeutic efficacy. Immunocombatibility between donor and recipients can reduce effectiveness of MSCs. MSCs typically exist as immune privileged cells due to their low expression of HLA-I and MHC-I (4). However, all cell modification strategies and culture methods can cause increased expression of immunogenic molecules. Furthermore, the inflammatory environment in recipient tissue such as interferon gamma can yield heightened MHC-II expression in donor MSCs, which can result in rejection and thereby necessitate repeated injections to achieve clinical benefits (49). The chemokine expression profile in recipient tissue can also be significantly heterogeneous and may not be compatible with unmodified MSCs. For example, areas of myocardial infarction may typically have high levels of expression of chemokines such as CXCL1, CXCL2, and CCL7; however, the presence of their corresponding receptors such as CCR1 and CXCR2 on native MSCs is low and can impede their delivery to such sites (50). As a result, careful molecular profiling of host disease tissue sites is needed prior to selecting targets to modify in donor MSCs to ensure maximal migratory and therapeutic potential is preserved.

The effect of the disease process within recipient tissue is also a major host factor limiting the efficacy of MSC therapy. For CVD such as myocardial infarction and PVD, significant ischemia and hypoxia in tissue results in a limited nutrient supply and hostile tissue interface for delivered MSCs to survive and proliferate in even after they have been modified. Moreover, patients with these diseases often have major comorbidities such as diabetes mellitus, smoking, and renal disease which can exacerbate ischemia and reduce the functionality of MSCs such as from hyperglycemia and circulation of uncleared uremic breakdown products (10). The utilization of biologic scaffolds and hydrogels can help provide a more hospitable cellular environment to areas of local delivery to improve the survival and retention of cell therapy. However, these methods to implant such scaffolds can require invasive surgical or percutaneous procedures to access cardiac and other internal organ sites thereby increasing the risk of infection and anesthestic complications in a patient population already predisposed to higher rates of complications (10).

## 7. Conclusions and future perspectives

CVD continue to be the leading cause of morbidity and mortality globally and claim the lives of over 17 million people annually (51–53). Current management of CVD includes risk factor modification and preventative strategies such as dietary and lifestyle changes, smoking cessation, medical management of hypertension and cholesterol lipid levels, and surgical revascularization interventions if needed (17, 54, 55). Although these strategies have shown therapeutic efficacy in reducing major cardiovascular events such as heart attack, stroke, acute limb ischemia, sequelae of CLTI, and major limb amputations, significant compliance by patients and caregivers is required and off-target effects from systemic medications can still result in organ dysfunction (17, 54). Given the rising incidence of CVD globally, newer strategies to reduce inflammation and stenosis associated with atherosclerosis progression beyond those imparted by conventional medical and surgical options are needed (52, 53).

MSC-based therapeutics hold substantial potential for tissue and vascular regenerative applications for cardiac and peripheral ischemic disease applications given their natural capacity for immunomodulation, self-renewal, and differentiation. However, delivery of sufficient quantities of cells to target disease sites without invasive intracoronary or direct injection methods remains a substantial challenge (10, 28). Methods to augment targeted cell delivery after systemic injection without indiscriminate trafficking to cell-sink organs such as the lungs and liver are critically needed for patients with these diseases. Recent preclinical advances utilizing physical methods to install adhesion moieties with nanocarriers or device-assisted induction of cell trafficking are avenues with emerging potential. Genetic methods have been widely applied to modify MSCs for other disease applications and are showing promise to enhance MSC phenotype and migratory capacity for cardiovascular applications. Several recent studies have demonstrated chemical modification strategies to influence the trafficking of MSCs to ischemic disease sites.

As investigators begin to translate these and similar preclinical advances into the clinical arena, several important considerations should be taken into account. Modification of MSCs by any strategy should include careful cell phenotypic characterization studies to ensure that innate stem cell properties including multilineage differentiation, self-renewal, and immunodulatory functions are preserved (56, 57). Nonbiologic nanocarrier platforms as well as genetic manipulation strategies utilizing viral vectors should be thoroughly interrogated with regards to their toxicity and safety after systemic administration of cell therapeutics (41, 58–60). Moreover, the cost and scalability utilizing any of these MSC modification domains should be considered prior to initiating preclinical and clinical investigations (48, 61).

Although this review focused primarily on the benefits of targeted MSC therapies, the technologies implemented to modify cells for homing to disease sites are extremely versatile and can be applied to the delivery of pharmacologic agents or additional cell types such as mononuclear cells and others (17, 62). Some investigators have modified mononuclear cells with adenoviral constructs to overexpress proangiogenic factors such as vascular endothelial growth factor (VEGF) and fibroblast growth factor (FGF), which results in their ability to induce endothelial cell proliferation and may represent future treatments for ischemic diseases (63). Moreover, future work may uncover that combinations of these aforementioned physical, genetic, and chemical alteration methods could produce the optimal cell phenotype to achieve localized cell homing to diseased tissue sites. Despite the challenges and limitations that will need to be overcome in translating these scientific advances into actionable clinical therapeutics, targeted cell delivery of MSCs holds tremendous potential and hope for patients with a wide variety of ischemic cardiovascular diseases that fail or are not candidates for other medical and surgical treatment options.
